# Somatic Experiencing^®^ Informed Therapeutic Group for the Care and Treatment of Biopsychosocial Effects upon a Gender Diverse Identity

**DOI:** 10.3389/fpsyt.2018.00053

**Published:** 2018-02-27

**Authors:** Paul C. Briggs, Sage Hayes, Michael Changaris

**Affiliations:** ^1^Healing Concepts, LLC, Hollywood, FL, United States; ^2^Embodied Liberation, South Portland, ME, United States; ^3^Integrated Health Psychology Training Program, Wright Institute, Berkeley, CA, United States

**Keywords:** social injustice, Somatic Experiencing^®^, transgender, discrimination, resilience, groupwork

## Abstract

**Background:**

Somatic Experiencing^®^ (SE™) is a resiliency-based treatment for autonomic nervous systems dysregulation syndromes, such as posttraumatic stress disorder, anxiety, depression, and physical syndromes like chronic pain, migraines, and fibromyalgia. “Transgender/gender non-conforming/gender variant” describes people whose gender identity/expression is different, at least part of the time, from the sex assigned at birth. Research indicates transgender individuals have a higher incidence of depression, anxiety, victimization, and discrimination. SE™ tools may support transgender/gender non-conforming individuals to increase resilience in the face of discrimination and social injustice.

**Methods:**

This study is a pretest posttest within group (*N* = 7) pilot study assessing the impact of a 10 session SE™ based group treatment on depression (PHQ-9), anxiety (GAD-7), somatic symptoms (PHQ-15), quality of life (QoL) (WHOQoL-BREF), and coping with discrimination (CDS) for a cohort of seven individuals identifying as transgender/gender non-conforming. Materials were created in collaboration with members of the LGBTQIA community. Care was taken to be inclusive of gender non-conforming identities and culturally responsive in design.

**Results:**

Participants described their gender identities as: non-binary, female to male, male to female, and gender fluid. Participants had significant increase in psychological QoL (psychological well-being) (WHOQoL-BREF) *p* = 0.004, SD = 2.31, with a modest effect size of *d* = 0.71. Some likely impacts of historical effect discussed. No other clinical or QoL outcomes were statistically significant. However, one outlier was identified in the dataset. When this outlier was excluded there was a trend toward significant reduction in depression symptoms (PhQ-9) *p* = 0.097, SD = 3.31 and a modest effect size of *d* = 0.68; somatic symptoms (PhQ-15) *p* = 0.093, SD = 3.52 and a modest effect size of *d* = 0.72.

**Conclusion:**

These data indicate that a brief 10 session intervention of SE™ could have a meaningful impact on symptoms of depression, somatization, and QoL for gender non-conforming individuals. Further research is warranted.

**Limitations:**

First, this study has a small sample size limiting statistical power and generalizability. Second is a history effect. Less than 1 week prior to final data collection, there was a significant hate-motivated act in Florida targeting the LGBTQIA community.

## Introduction

Somatic Experiencing^®^ (SE™) is a resiliency-based treatment for autonomic nervous system (ANS) dysregulation syndromes, e.g., physical syndromes like migraines, chronic pain, irritable bowel syndrome, chronic fatigue, and fibromyalgia, as well as anxiety, posttraumatic stress disorder (PTSD), and depression. SE™ focuses on re-establishing an individual’s innate capacity for ANS, physical, and emotional regulation. According to SE™, trauma resides in the nervous system, not in the traumatic event itself, and is a relatively short-term, somatically based approach to resolving trauma (1). The results of a study of 150 victims exposed to the swift impact of a Tsunami revealed that even 8 months after the event, trauma symptoms, such as intrusion, arousal, and avoidance, either improved or were eliminated for victims provided with an SE™ intervention shortly after the event (2). In the first randomized controlled study of the effects of SE™ for the treatment of PTSD, researchers Brom et al. (3) concluded that SE™ may be an effective therapeutic method for PTSD. They studied the outcomes of the intervention effects for posttraumatic symptoms severity and depression among 63 participants who received 15 weekly SE™ sessions.

It is well documented that a more severe symptom profile with respect to pain, disability, and psychological distress stands comorbid to PTSD in chronic pain. In a first-ever two-group randomized controlled clinical trial evaluating the effect of SE™ for treating comorbid PTSD and low back pain, a cohort of 1,045 patients was studied by researchers Andersen et al. (4). The authors found that SE™ intervention significantly reduced the number of PTSD symptoms and fear of movement, and both comparison groups achieved a large reduction in pain catastrophizing, disability, and pain. Thus, a brief additional SE™ intervention was found to have a significant effect on PTSD and fear of movement compared to a group receiving only standardized treatment. Researchers add that the clinical importance of the effects can be questioned, as the overall effect of SE™ was less than expected.

Payne et al. (5) indicate that SE™ is not a form of exposure therapy. SE™ differs from cognitive therapies in that the strategy utilizes a bottom-up processing of limbic and brain stem activation states rather than a top-down or cognitive regulation. SE™ technique guides attention toward interoception of visceral states and proprioception/kinesthesis of musculoskeletal position and tension. The authors explain that SE™ initially works with a focus on a gradual increase of resiliency, working in a titrated manner to explore traumatic affect, and memories. Using this method of gently, gradually, and indirectly approaching charged memories allows for the development of empowerment in managing the intensity of traumatic states. Ultimately, positive experiences of mastery/empowerment are generated, contradicting the negative traumatic state of overwhelm. These new possibilities likely overwrite the traumatic event’s outcome with the generation of corrective interoceptive/kinesthetic/proprioceptive experiences, the authors convey.

### Gender Identity and Gender Dysphoria

A psychiatric condition termed Gender Identity Disorder was a former label for individuals who experienced a significant incongruence between their gender identity, or sense of being male or female, and their physical phenotype, resulting in a chronic suffering known as Gender Dysphoria (6). Gender Dysphoria as defined by the American Psychiatric Association in the DSM-V (7) became the revised diagnosis relative to a marked incongruence between one’s experienced/expressed gender and assigned gender. In a study involving the review of medical charts of 435 gender dysphoric individuals, Cole et al. (8) recognized that gender dysphoria is usually an isolated diagnosis, and individuals experiencing gender dysphoria do not appear to have problems indicative of coexisting psychiatric illness, such as major depression or schizophrenia. Once the individuals studied were able to acknowledge their gender dysphoria, they were able to perceive themselves as happier, more competent, and they felt more productive in vocational and other activities.

Current treatment recommendations for and relief from Gender Dysphoria symptoms, within a male-female binary context, involves a transitioning process toward the desired experienced gender (9). This process includes cross-sex hormone replacement therapies (HRT) and can also involve an array of gender-affirming surgeries intended to change one’s body to achieve the physiological look, feel, and function of the body in order to conform to one’s gender identity. For many, these surgeries can be cost prohibitive to acquire, and sometimes dangerous when attained under sub-standard, less costly means. In a 5-year follow-up study of 19 transsexuals in different phases of a change-of-sex process, Bodlund and Kullgren (10) found that 70% improved in different social, psychological, and psychiatric aspects. This supports fairly positive outcomes in several important areas for these transsexuals changing sex. Gómez-Gil et al. (11) realized that 67 individuals out of 187 transsexual patients on a gender identity unit who had not initiated cross-sex hormonal treatments reported higher levels of social distress, anxiety, and depression. The remaining 120 who had undergone hormonal sex-reassignment treatment reported subclinical levels of social distress, anxiety, and depression.

While much of the literature reviewed supports more favorable views of self-image once HRT and reassignment surgeries are achieved, Devor (12) proposes a biopsychosocial model which supports a more comprehensive and inclusive consideration of transsexual identity formation. The term “transsexual” has been used to define those people who self-identify within this binary context (e.g., gender as a binary of male or female). The broader, more widely preferred term “transgender” describes a person (or even a group of people) who do not fit into the binary context of gender categories of male and female (13). “Transgender/gender non-conforming/gender variant” describes people whose gender identity or expression is different, at least part of the time, from the sex assigned to them at birth. Gender nonconformity is not the same as gender dysphoria, and only some gender non-conforming people experience gender dysphoria at some point in their lives (9).

A view of gender spectrum from a health-based perspective would not be rooted in a health or mental health disease model. However, some transgender individuals tolerate the insinuation of transgender identities stemming from a biological or mental health disease origin in part due to the economic realities of current health-care delivery. Many fear that activism and advocacy efforts to remove “transgender labels” associated with “disease,” will diminish the vital importance of the need for hormones or gender-affirming surgeries, which require a diagnostic label in order to be paid for by insurance companies, while others desire to be free from imposed labels (14).

Markman (15) proposes that the distress experienced by transgender and gender variant people is not the fault of some individual pathology but is instead the result of problems generated by a society that perpetuates ignorance, prejudice, and bigotry. “There is no one way to be transgender.” Exposure to discrimination in the social context has an impact on health and mental health overall. People who identify as transgender come from all professions, income levels, sexual orientations, age groups, races, and live in every country in the world. Transgender individuals may define their gender identity as their inner sense of being male, female, both or neither. Transgender people often vary on how they describe and identify themselves relative to their backgrounds, where they live, who they spend time with, and how the media influences their self-definition, resulting in an ever-changing terminology that best fits and describes who they are (16).

### Gender Identity, Community Violence, and Microaggressions

Transgender persons within the US are marginalized in many ways due to discrimination and various other psychosocial barriers. From a 70-question survey of over 7,500 people, the largest and most extensive study of its kind to date, Grant et al. (17) indicate that the transgender population reports a higher incidence of depression, anxiety, mental health concerns, suicide, lack of access to health care, unemployment, violence, victimization, and discrimination. Much of the literature reviewed supports this indication. Additionally, transgender persons must live their daily lives in a world constantly challenged by social expectations regarding their gender expression and presentation (18). In an exploratory study by Nadal et al. (19), the authors indicate how overt forms of bias and discrimination, such as hate crimes, systemic oppression, and injustice, affect transgender and gender non-conforming people. Sources of bias and discrimination include health care, family, employment education, the criminal justice system, and other public accommodations and service providers. The authors recognize that more subtle forms of discrimination, known as microaggressions, can also have detrimental effects on individuals belonging to the transgender community. Microaggressions, which consist of microassaults, microinsults, and microinvalidations, come from various environments across life domains, including educational systems, workplace settings, the media, and the general community (20). These exposures can have a negative effect on mental health and well-being. A study of 571 male to female (M → F) transgender individuals confirms the prevalence of high exposure to psychiatric distress and gender-related abuse over the course of a lifetime, as is indicated in much of the literature. In this study, Nuttbrock et al. (21) found that 78.1% of study respondents previously experienced gender-related psychological abuse, and 50.1% previously experienced gender-related physical abuse, supporting a causal association between gender abuse and depression or suicide.

Studies have confirmed, over the past two decades, that there are consistent associations between exposure to discrimination and mental and physical health, with an impact upon a wide range of DSM mental disorders as well as objective physical health outcomes (22). Because of the way they are treated by society; minority, oppressed, or marginalized groups are at greater risk for experiencing mental health issues, according to the minority stress theory. Transgender people face discrimination both within the lesbian, gay, and bisexual community, and within society as a whole, and gender-non-conforming individuals often belong to other oppressed groups as well. Increased anxiety is also an experience of many transgender and gender non-conforming people who are exposed to high levels of violence, victimization, and trauma (23).

### Somatic Experiencing^®^ Model and Exposure to Discrimination

Chronic exposure to discrimination and stress in one’s social contact can lead to intense feelings of stress as well as at times feelings of overwhelm and being out of control. According to Scaer (24), “almost any social setting where control is lost and relative helplessness is part of the environment can easily progress to a traumatic experience.” During a traumatic or threatening event, lower, more primal brain centers become engaged, and executive higher brain functions become less active. A more dominant hard-wired neurological reaction occurs involving the reactions of orienting, fight, flight, or freeze (2). A person’s reactions can become conditioned to aspects of this life-threatening event as a traumatic experience, and subsequent exposure to similar events can trigger an involuntary portion of the terror reaction in the body to be replayed (25). SE™ (26) works to gently gain access to these involuntary responses, build the person’s awareness of the bodily reactions, and actually “process” the reactions to an “adaptive resolution.” When an event creates an unresolved impact on an organism, trauma occurs. By working with the felt sense, resolution of a traumatic impact can be achieved. Traumatic stress reactions, when left untreated, have been found to often result in long-term negative mental health effects (27–29).

The work of Levine (30) reveals that the “freeze response,” a state of immobilization associated with individuals who experience trauma, can manifest into numerous debilitating somatic symptoms, including dissociation, feeling trapped and helpless, shutting down, and numbing. When the sympathetic nervous system is activated in a heightened state, the parasympathetic nervous system also becomes activated, resulting in a slowing or shutting down of body systems. The resulting numbing can create a dissociation, where people are no longer “in” their bodies. Many people who become traumatized find that the experience of “being in their bodies” can in and of itself feel unsafe and frightening (31). Left unresolved, this immobility reaction becomes chronically coupled with intense negative emotions, such as dread, revulsion, and helplessness. A chronically coupled fear becomes paramount, and this now conditioned fear perpetuates into a traumatized person remaining fearful of even internal (physical) body sensations, which then generates even more fear, further deepening the paralysis or “freeze,” forming a type of trauma vortex (30). Leitch et al. (31) indicate that in addition to psychological trauma, substantial evidence exists that those who survive trauma also suffer significant and debilitating physical or somatic symptoms relative to their experience. Getting in touch with the process of the traumatic response, rather than recreating and reliving the event, is the key to healing (32). Body psychotherapy techniques that incorporate body psychotherapy, such as SE™, promote the emotional release and psychological stability by correcting hyper-arousal in the person’s physiology, immobility, and ultimately re-stabilizing the ANS (33).

Hopwood and dickey (34) encourage members of the transgender community to pay attention to their health and well-being. Mental health professionals can assist with ways to relieve stress, provide more information in order to discover ways to confirm and express gender identities and help people to manage interruptions or difficulties in life, which can create sources of distress, even in a relatively balanced life. It is possible for individuals to regain balance in mental health by being pro-active in locating their own supports and services. Therapists who are competent in working with transgender and gender non-conforming individuals are increasing in numbers and offer a variety of therapeutic relationships through individual and group experiences.

### Aim and Significance

This study represents a proof of concept study that explores the possibility that a 10-week SE™ informed psychotherapeutic and psychoeducational group experience can establish a group work model to foster resiliency toward the management and possible resolution of symptoms of distress related to Gender Dysphoria; as well as related to discrimination and other psychosocial barriers faced by Transgender, Gender-Non-Conforming, and Gender-Variant individuals.

## Materials and Methods

### Overview

This study is a pretest posttest within groups design. Selected Transgender, Gender-Non-Conforming, and Gender-Variant individuals were assessed on changes resulting from particpation in a 10-week SE™ informed psychotherapeutic and psychoeducational group experience. There were two points of measurement: at the start of the group sessions, and again after completion of participation in the 10 weeks of sessions. This study was carried out in accordance with the recommendations of the “IRB Committee at the Wright Institute” in Berkeley, CA, USA, with written informed consent from all subjects. All subjects gave written informed consent in accordance with the Declaration of Helsinki. The protocol was approved by the “IRB Committee at the Wright Institute”, and it is identified with the Wright Institute as “SE Transgender Care Research Protocol (IRB) r05-03-15 FINAL.”

### Measures

Significant studies indicate that the Transgender population reports a higher incidence of depression, anxiety, mental health concerns, suicide, lack of access to health care, unemployment, violence, victimization, and discrimination. Additionally, Transgender persons must live their daily lives in a world constantly challenged by social expectations regarding their gender expression and presentation, causing undue stress and anxiety.

The Patient Health Questionnaire-SADS (PHQ-SADS) (35) is a brief measure with strong validity and reliability of stress, somatic symptoms, anxiety, and depression. The World Health Organization Quality of Life (QoL)- BREF (WHOQoL-BREF) (36) is a clinically sensitive measure of quality with solid validity and reliability. It measures four QoL domains (psychological well-being, health-related well-being, social relationship-related well-being, and environmental well-being). The measure is flexible and was developed to be adaptable to multiple settings and populations. Psychological symptoms as measured by PHQ-SADS and wellbeing as measured by WHOQoL-BREF are correlated with resilience. There is a large body of research and literature on the relationship between QoL and management of anxiety and coping with stress. Originally developed for use with cultural, racial, and ethnic minorities experiencing discrimination, the Coping with Discrimination Scale (CDS) (37) is a brief measure that has solid validity and reliability as a measure of coping factors relative to the experiences of discrimination. The language context appears to transpose appropriately to evaluate coping of Transgender persons experiencing discrimination in multiple dimensions of their life experiences.

### Statistical Analysis

This is a pretest, posttest within groups design. Main effects will be assessed using paired one-way *t*-Tests for each variable and significance will be reported at a *p*-value of 0.10. All comparisons groups that do not conform to assumptions of within groups *t*-Test, i.e., does not meet criteria for normal distribution will use Wilcoxon single rank *t*-Test. Pearson’s *r* will be used to assess the correlation between CDS scores and outcomes on PHQ-SADS and WHOQoL–BREF. Normality of distributions will be assessed using visual analysis on Q–Q plots as well as Sharpiro–Wilk test. Data that do not conform to the assumption of Person’s *R* will be assessed using Spearman’s Rank. Data will be assessed for outliers using visual assessment *via* scatter plot, SDs from the mean, and the Dixon outlier test.

### Participant Initial Recruitment and Selection

A convenience sample of adult group participants who may be experiencing and/or have been formally diagnosed with Gender Dysphoria was solicited from within the South Portland, ME, USA Transgender community. Participants needed to self-identify as “Transgender/gender non-conforming/gender variant,” which describes people whose gender identity or expression is different, at least part of the time, from the sex assigned to them at birth. They could also be in a process of transitioning from their physiological gender to their self-identified gender in a binary context. Individuals who passed pre-screening were given the opportunity to participate in a 10-week SE™ informed group therapy intervention model.

**Figure 1:**
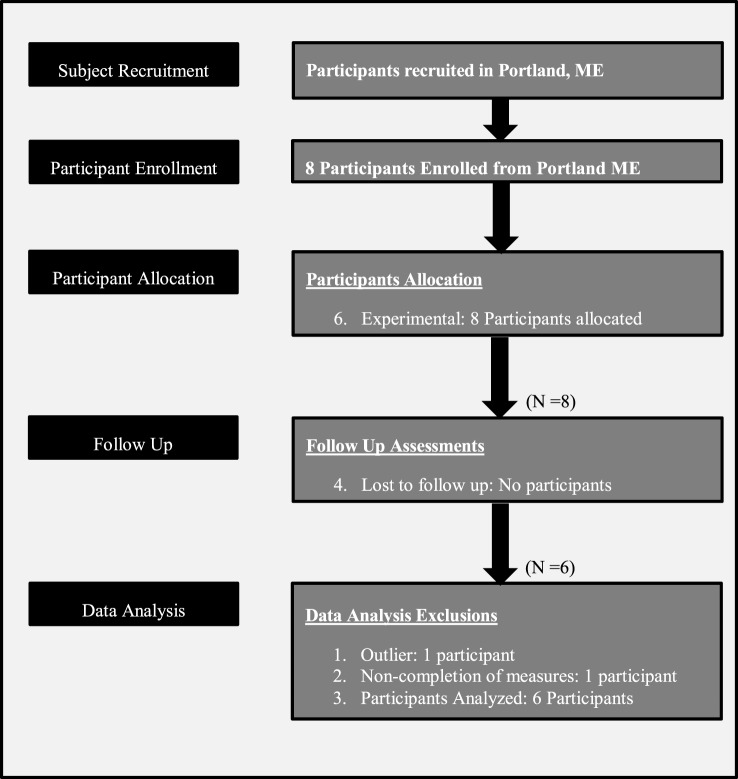
Participant flow.

Appropriate candidates were provided with additional instruction at an informational meeting. During this event, candidates had an opportunity for discussion of the procedures, the risks, and benefits of participation in the study, their rights as a research participant, confidentiality procedures for their personal information, time commitment, group schedule, and duration. Participants were informed that treatment referrals were available should they have an adverse reaction to the testing or else discover a need for care. All went through a three-level informed consent process and completed a written informed consent acknowledgment. They were apprised that any data collection is private, the group experience is confidential, and no personal data would be retained that could connect an individual with their measures. The signed informed consent forms and all subsequent collected data were stored separately, maintaining appropriate HIPAA compliance at all times. Participant flow is represented graphically in Figure 1.

### Initial Group Participant Establishment Challenges

The original design of this study was to consider the results of a comparison between two independent group experiences with groups running in two different geographical locations. Group parameters and curriculum would be held to consistency, and each group would be facilitated by a different author. Two well-established community locations equally offered a safe, consistent, familiar meeting space, ancillary participant support, as well as a source for soliciting participants. One location was to have been at Sunshine Social Services (SunServe), a local Gay, Lesbian, Bisexual, and Transgender + Social Service Agency located in Wilton Manors, FL, USA. The second location considered was Embodied Liberation in South Portland, ME, USA.

Transgender support group promotion began in South Florida. Palm cards were printed, and distribution and community visibility occurred over a 6-month period at several major Transgender events significant to the local Transgender community. The hosting agency, SunServe, provided additional promotion through word of mouth and social media information distribution on their agency website and Facebook page. At the end of the promotional period, only 10 prospective participants expressing interest. Despite these group promotion efforts, we were only able to finally contact, screen, and identify 5 individuals for the group, from a total of 10 inquiries. Five of those who had expressed interest early on were no longer available once the group was ready to begin.

The five identified individuals were contacted by telephone, and through e-mail and were provided with the exact dates and times of the introductory meeting and the 10 subsequent support group dates. On the evening of the introductory meeting, none of the five individuals attended, nor did they make contact with the facilitator to advise that they could not attend.

We, along with SunServe staff, met and discussed possible reasons for a lack of turnout on the first evening of the group. Possible South Florida site factors included:

– during the day prior to and the day of the initial group evening, weather in South Florida was rainy, damp, and there had been an unusual threat of tornado touchdowns in the area.
– participants in this group may need additional reminders, i.e., phone call/e-mail just prior to the date of the group.
– the group facilitator identified as a gay, cisgender male.
– although the facilitator has been involved in the South Florida LGBTQ Community for over 18 years, he is not well known in the Transgender Community.
– the group facilitator had only been able to make telephone and e-mail contact with participants, there has not yet been an opportunity to meet face to face.

Our study was finally conducted using only one group, without an opportunity for comparison, with participants selected from the South Portland, Maine community. Group members were solicited from the community in a similar fashion, through palm card distribution, social media, and word of mouth. Eight participants were identified and engaged in the study. Positive site factors considered included:

– the group facilitator identified as a Transgender person.
– the facilitator is a member of the South Portland Transgender Community.
– the facilitator is a practicing massage therapist in the South Portland Community for many years.
– the facilitator has an established relationship and is well known and embedded in the community.

### Administration of Measures

Selected candidates were given a packet of measures consisting of a demographic survey, the PHQ-SADS, the World Health Organization QoL–BREF (WHOQoL-BREF) scale, and the Coping with Discrimination Scale (CDS). The same set of measures, with the exception of the demographic survey, was administered once again at the conclusion of the 10 weeks of sessions.

### Somatic Experiencing^®^ Informed Group Therapy Intervention Model design

#### Group Therapeutic Goals

The intention of the group is to assist participants who may be experiencing Gender Dysphoria to develop: a general increase in awareness of resources, skills, and abilities, increased skills for affect management, increased skills for nervous system regulation, increased capacity for tolerance of “being in their bodies” and managing “real life” situations relating to confrontations and microaggressions, increased resiliency, a decrease of negative symptoms such as depression, anxiety, and feelings of social isolation. Subjects who participated in the study may have experienced discomfort while answering some pre- and posttest questions, and during participation in a therapeutic group process. But there is no clear data indicating iatrogenic effects of answering the mentioned measures. Further, subjects may have experienced discomfort relative to the emergence of material relative to their therapeutic process during their group experience. The group setting is intrinsically designed to be a safe, supportive environment to nurture growth and change. Heck (38) indicates that the efficacy of services for the care of transgender/non-conforming individuals has not yet been established, however, there is a small but growing body of scholarly work focusing on competencies necessary for the delivery of quality transgender-affirmative care. As the needs and challenges of transgender/non-conforming individuals are further identified, work can evolve from writing about providers’ group work experiences with this population, to actually empirically evaluating the efficacies of and implementing interventions that demonstrate positive identity-related and health outcomes. This is one goal of our work. In a study using a brief (one to two session), SE™ informed Trauma Resiliency Model intervention, Leitch et al. (31) found that the resiliency of participants increased, while psychological distress was found to significantly decrease. Adequately addressing trauma experiences from self-directive, interpersonal, and collective perspectives, utilizing both idiographic and nomothetic approaches to clinical practice was found by Richmond et al. (39) to be of importance. A single, 75-min treatment approach used by Parker et al. (2) focused on the introduction of psychoeducational information related to the involuntary nature of trauma symptoms and on participant practice subsequent to the intervention, yielding a reduction or elimination of trauma symptoms for 150 participants, even after 8 months. This indicates the effectiveness of their protocol on dysregulation from trauma. Our SE™ informed group design sought to utilize these ideologies.

#### Prescreen for High-risk Behaviors and Group Appropriateness

Maintaining the ongoing safety and support for participants at all times was an utmost priority in this study. A set of screening questions was developed and utilized to identify those individuals whose capacity for managing these possible periods of discomfort and shifts in cognitive awareness is compromised by a history of high risk for self-harm, severe mental illness, or active use of mind-altering substances. For these individuals, participation in the study was not recommended. Group members who were selected to participate were asked to not be under the influence of alcohol or other mind-altering substances during participation in groups.

#### Group Makeup, Setting, Location, and Facilitation

Several additional factors were considered for the design, creation, and implementation of a therapeutic group model that could be both extremely effective for as well as hold the interest of participants. Such factors included the safety of the setting, both actual and perceived, group facilitation, program consistency, a high degree of being SE™ informed, access to ancillary supports outside of the group experience, and ease of replication.

The group met weekly, in 10 consecutive 90-min sessions, was free of charge to participants, and was offered to appropriately screened adult individuals aged 18 and over who identified as transgender, gender non-conforming, or gender variant. Participants could also have a formal diagnosis of Gender Dysphoria, which was not a requirement for inclusion. We chose to impose an age restriction upon our participant sample, solely to avoid the need to obtain parental consent for participants under 18 years of age. We firmly believe that the design and benefits of an SE™ informed Transgender Support and Healing Group experience would be equally appropriate and effective for young adults and adolescents, and with some modifications could be effective for working with children as well.

It was important to provide group participants with a consistent, appropriate, safe, and familiar space to meet each week. Having the perception of a predictable and safe environment is crucial for participating individuals, as this has been shown to aid in adjustment in a variety of traumatic situations. It is important to have knowledge of the many ways intersecting oppressions can inform participants’ perceptions of a predictable and safe environment (40–42). The initial development and ongoing maintenance of a safe environment both inside the therapy room and at large as well is of great importance (43, 44). Within the context of SE™ informed work, it is certain that group members’ nervous systems will respond in understandable and predictable ways, as the group experience touches on situations of stress, trauma, overwhelm, and interpersonal risk. These very same responses occur in the daily lives and experiences of group members, in relationships, in the workplace, as well as in the therapy group. The group setting affords a container where members can discover or recover the opportunity for self-regulation (45).

Participants were solicited from and the group was purposely held within the same South Portland, ME, USA Transgender community. Group facilitators were clinicians or practitioners with a professional level of training, experience, and expertise regarding group work process and SE™ work, in our case one of us. The role of the primary group facilitator was to guide the group through the SE™ informed curriculum and associated exercises, utilizing their skills as a practitioner. As well, the primary group facilitator works at tracking the nervous system at multiple levels, that of individuals, and that of the collective nervous system of the group. The facilitator provides support for the group and individuals, as the capacity to tolerate more intense experiences increases, within the range of resilience (45). An optional second care provider could be a student or intern from a social work, mental health, or bodywork background. Their role would be to monitor group members, recognize nervous system activation or overwhelm, and be able to intervene with support for group members when needed. A second care provider was not utilized in our case.

#### Agency Staff/Clinician Ancillary Support and Education

Subjects who participated in this study may also have had the added benefit of already being engaged in other pre-existing relationships of supportive psychotherapy and/or case management, by virtue of their possible affiliation with community providers or agencies. Otherwise, they did not receive any unique or additional treatment, procedures or information other than the participation in the group experience and data collection.

Clinicians and staff of affiliated community agencies can play an integral part in providing participant support outside of the group setting, as participants work on change in the group process. Considering this, prior to the start of the group series, local community agency providers were offered an orientation regarding: concept and principles of SE™ work, what will be done in the group setting, predictable outcomes and responses of participants making change during their process, ways that providers can be supportive allies during this work. The option was given for participants to grant written permission for individual providers and group facilitators to be able to communicate regarding any emerging concerns relating to group members’ participation in both individual and group therapy sessions.

### Curriculum Design Considerations for Somatic Experiencing^®^ Informed Work

The foundation of the group experience is based upon Somatic Experiencing^®^, a well-recognized treatment model for working with dysregulation syndromes such as PTSD, anxiety, and depression; focusing on re-establishing an individual’s innate capacity for ANS, physical and emotional regulation. SE™ informed work involves the application and integration of SE™ theory, concepts, and practice principles into a treatment model; in this case a Transgender Support and Healing Group experience. The goal being to address the care and treatment of and give attention to biopsychosocial affects upon a gender diverse identity which can contribute to some form of Gender Dysphoria. This study targets how SE™ informed work, and more specifically working with the “felt sense,” can assist Transgender, Gender-Non-Conforming, and Gender-Variant persons with alternative ways to help manage, re-establish, and build capacity for ANS, physical, and emotional regulation. There is a body of literature that indicates increased skills in self-regulation leads to increased resiliency and coping. In the interpersonal process of an SE™ informed group setting, the survival physiology and activation patterns related to fight, flight, or freeze responses can be re-worked *in vivo*, allowing for the alteration of interpersonal symptoms often created from these responses (45).

#### Group Format: Structure, Content, and Flow

Design consideration for this study consisted of learning and SE™ skills building assembled into 10 themed modules, which included the presentation of new material and concepts, reinforcement of learned concepts, overlap, and building upon skills acquired in previous sessions. Experiential exercises that familiarize participants with and engage the “felt sense” were incorporated. As well, exercises that helped group members to learn about and practice regulation of the sympathetic and parasympathetic branches of the ANS were implemented into the core of skills building. Addressing ways to practice self-care was also vital as a means to reinforce new material and skills.

The structure of each module consisted of title, content/activities, intention, target issues, skills taught, homework, and supplies. We built in consistency to each module design, so that appropriate time could be allotted for necessary group functions. For each 90-min session, we allotted: settling/grounding—5 min, check-ins—15 min, content (teaching, experiential opportunities, integration of information, and experiences)—55 min, and wrap up/closing—15 min. More detail is provided in Table 1: Somatic Experiencing^®^ Informed Transgender Support and Healing Group Module Structure.

**Table 1:** Somatic Experiencing^®^ Informed Transgender Support and Healing Group Module Structure.

Module Title	Content/Activities	Intention	Target Issues	Skills Taught	Homework	Supplies
1. Grounding (welcome)	Please consult with authors for details ↓	Have participants share why they are here and why we are here as Trans people Set agreements Intention setting Set tone, Grounding - physical/energetic space	Safety/Trust Grounding Self-Expression	Group sharing Learning to make agreements with each other Working collectively	➢ Trans History and Timeline-bring in at least 3 significant events	

2. Resourcing and Trans History Timeline		Framing and introducing embodiment, resourcing, felt sense Visibilize Trans history Utilize Trans history as a resource of empowerment and visibility	Self-awareness Increasing understanding Historic and multi-generational empowerment in the body Self-esteem	Tracking felt sense Reflecting on life and history in a historical and resourcing way	➢ Bring picture of significance representing own personal process regarding gender, expression, and identity	Visuals for Trans history (optional), markers, tape, paper for timelines

3. Campfire. Mindfulness. Awareness. Tracking		Deepening connection, cohesion, trust Sharing personal stories/identities Generate a “lived” definition of what Trans is to our group	Self-esteem-Highlighting personal and collective resilience amidst challenges, coping, surviving Building positive personal and collective identity Introduction of tracking/paying attention to the body	Felt Sense Tracking Resourcing Reflection and sharing Valuing of diversity of experiences, identities and expressions	➢ Check in with one person within the group over the week	large bowl, candles for “campfire”, s’more sticks, marshmallows, chocolate, graham crackers, bucket of water for safety, chime

4. Nervous System Overview and Resilience		Teach about nervous system and trauma 101	Regulation/dis-regulation Orienting Understanding and self-awareness	How to track the threat response cycle and complete it	➢ Tracking felt sense	SE™ skills visualsSE™ Powerpoint or posters

5. Safety. Boundaries. Protective Mechanisms		Explore and understand embodied safety Collectively define the different types of safety in different parts of life	Name transphobia as contextualized (social) trauma Geo-macro-micro Empowerment Safety inventories	Experience of personal space Experience of tracking where personal boundaries are Boundary setting	➢ Increase awareness of safe and unsafe spaces - people, places, things	

6. Dealing with the Stress of Microaggressions		Teach about and make visible microaggressions Teach about the impacts of microaggressions on the body Teach what happens in the body when it is under threat	Teach about and make visible microaggressions Teach about the impacts of microaggressions on the body Agency and response to microaggressions	Self and environmental awareness Detection of microaggressions Self-care for microaggressions		

7. Working with Depression and Anxiety Developing a Self-Care Practice		Centralizing self-care as essential Normalizing depression/anxiety as adaptive responses to chronic threat and stress	Compassion for self and others regarding depression/anxiety Self-care, self-regulation	Self-assessment of different aspects of health Self-care skill through actual self-care activity (foots soaks, etc)		

8. Building Trauma-Informed Caring: Trans Community and Support		Use awareness practices to support our capacity to stay with exactly what is going on	Building tolerance for activation Creating direct support for activation	Emotional and physical capacity building for sensations Support giving Support receiving Feeling tones		

9. Embodied Liberation, Thrive. Resolutions, Completions		Embodied Visioning - using SE™ concepts to imagine where you want to go	Empowerment Liberation Thriving Embodiment	Implementing skills and experiences into an action plan Envisioning a positive future		Paper, worksheets

10. Party, AppreciationPostTest		Wrap-Up group Identify learnings from past 10 weeks Mark what has changed over past 10 weeks	Completion Strong and healthy ending	Reflection Sharing		

#### Resourcing

Integral and vital to SE™ informed work is the concept of resourcing. Initial treatment must involve reinstating lost resources, learning new ones, and strengthening those that exist. This concept is fundamental in many body-based approaches and is essential when working with traumatized clients. Ogden et al. (46) explain that traumatized clients typically have unregulated nervous systems, a perceived or actual loss of safety, compromised functioning, and somatic, emotional, and cognitive confusion between the past and the present. These patients can become further destabilized unless interventions are introduced that provide stabilization and increase the capacity of the nervous system so that adaptive functioning in daily life is increased. Ogden et al. (46) define resources as “personal skills, abilities, objects, relationships, and services that facilitate self-regulation and provide a sense of competence and resilience.” Heller and Heller (47) indicate that “a resource can be any positive memory, person, place, action, or personal capacity that creates a soothing feeling in a person’s body.” Resources can be the parts of our bodies that feel good, or the past and present positive experiences in our lives. Accessing positive resources in the “here and now,” with a focus on what is working rather than what is not working for a client, has a therapeutic impact in three different domains: cognitive, emotional, and physiological (48). The authors indicate that cognitively, clients can recognize ways to shift their thinking away from self-blame and self-judgment, avoid shame, and become more self-accepting. Emotionally, clients frequently take for granted that they have survived adverse difficulties, with great courage and fortitude, for sometimes a very long time, and often lose sight of the fact that there has been and still is support for them through their internal strengths. Body-based resources in the here and now have the most power, as getting in touch with internal and external resources facilitates the capacity for nervous system regulation, producing a further calming effect.

We believe that the group setting itself can provide a source of resourcing for participating members. Much like the herds of animals that Peter Levine studied in the wild, a group can operate with a “collective nervous system,” sensing, identifying and responding to common threats that may evoke in the group process and dialog (30). Conversely, a group setting can also represent resources that provide a sense of safety and so can replicate the protective factors afforded by a herd, group members can resonate on the senses and resolution of the threat response, can find support among fellow group members, and so can collectively operate in the healing process. Group members can recover their innate sense of what is and is not safe and can vicariously learn for themselves through bearing witness to, resonating with, and managing distress evoked by the shared experiences of others in the group (45).

We believe that transgender history can be a powerful resource, as many transgender individuals lack a reference to positive role models, pioneers, “heroes,” and events in history, instead of being constantly reminded of a sense of insignificance and the negative challenges they must confront. We integrated a “trans history” exercise into our curriculum so that group participants could take positive and affirming ownership of some of the many notable origins of “being transgender.”

#### Social Engagement

Re-orienting in the here and now is an integral part of an SE™ informed process and being in the group presence can serve as part of this important function. The capacity for embodied social engagement is intrinsically self-calming and appears to make a powerful contribution to health and happiness, according to Levine (30). This capacity can provide a protection for the person, instilling their organism from being “hijacked” by the sympathetic arousal system, or else frozen by a more primitive emergency shut down system. The group setting provides a source of commonality for both grounding and normalizing of individual participants’ experiences.

#### Movement Interlude

At times, it would be necessary to assist the group with ways to collectively re-regulate participants’ ANS, especially after highly charged material emerged from within the group process. We developed a tool and coined it “Movement Interlude,” that could be used when group facilitators recognized high levels of group energy that may be too collectively overwhelming or too difficult for the group or a majority of individuals to manage. This tool is a physical movement intervention when the need for activation energy discharge is necessary. The possibility of use of this interlude and its purpose was previously discussed with group members, and a brief practice run occurred. One group member was invited to lead the group in a brief, creative random movement exercise (i.e.,: marching/walking in place, movement of extremities, conga line, etc.) that allowed the opportunity for collective and individuals’ energy to be discharged. The exercise would take approximately 2 min, with an additional 2 min for grounding, settling, centering, and re-focusing. During this time, group members were encouraged to share about what they noticed for themselves and for the group.

## Results

### WHOQoL-BREF

*N* = 7 participants had complete data for the WHOQoL-BREF. As shown in Table 2, significant improvements in QoL measures were observed in one of the four domains of the WHOQoL-BREF. Psychological QoL (Psych. QoL *p* = 0.003) was found to display both a statistically significant and a clinically meaningful improvement. This change would also represent a meaningful increase a participant’s ability to psychologically respond to stressful life events. The effect size for this change was moderate with a Cohen’s *d* = 0.71. All other domains were non-significant. The below data sets met criteria for normality using visual assessment of line on Q–Q plot as well as Sharpiro–Wilk test.

**Table 2:** Changes in quality of life (QoL) as measured by the WHOQoL-BREF (*N* = 7).

	Pretest	Posttest	*p* for model	Effect sizeCohen’s *d*
HEALTH—QoL	54.57 ± 26	61.43 ± 31.46	0.18 (NS)	–
PSYCHOLOGICAL—QoL	41.86 ± 20	58.29 ± 25.9**	0.003***	*d* = 0.71
SOCIAL—QoL	67.86 ± 25.1	66 ± 28.7	0.35 (NS)	–
ENVIRONMENTAL—QoL	56.3 ± 23.2	51.86 ± 21.7	0.22 (NS)	–

*Increased scores on all QoL measures indicate improved QoL*.

*Significantly decreased between pretest and posttest p ≤ 0.10.

**Significantly decreased between pretest and posttest p ≤ 0.05.

***Significantly decreased between pretest and posttest p ≤ 0.01.

*NS, non-significant results*.

### WHO-BREF-QoL Graphic Display of Data

**Figure 2:**
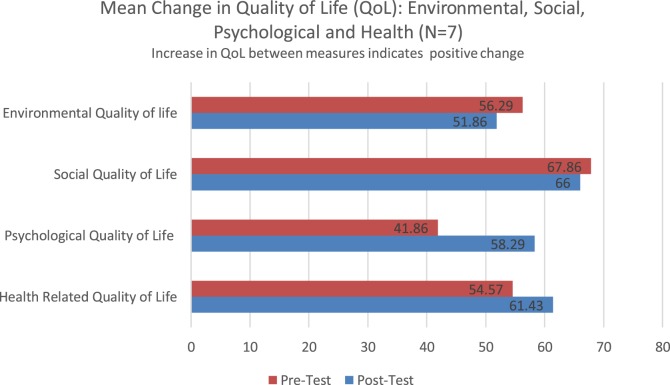
WHOQoL-BREF: environmental, social, psychological and health domains.

Figure 2 displays mean changes between pretest and posttest on four domains of the World Health Organization QoL measure. Increases in QoL score between measures would indicate a positive change. Both health-related QoL (mean increase of 6.96 points) and psychological QoL (mean increase of 16.43) showed improvement between measures. Social and environmental QoL showed small declines between measures with a mean reduction of QoL of 1.86 points and 4.43 point reduction, respectively. The results indicated a significant improvement in psychological QoL (*p* = 0.003) on the WHOQoL-BREF, effect size *d* = 0.71. The increase in psychological QoL would be clinically meaningful and likely would represent an improved overall psychological sense of wellbeing.

### PHQ-9, GAD-7, and PHQ-15 (Outlier Included)

*N* = 7 participants had complete data for three clinical measures. These measures assessed depression (PHQ-9), anxiety (GAD-7) and somatic symptoms (PHQ-15). None of these measures were significantly reduced. The data did indicate a reduction in clinical symptoms between pretest and posttest in all three measures. These changes could have a modest impact in an individual’s overall functioning. Data sets shown in Table 3 met criteria for normality using visual assessment of line on Q–Q plot as well as Sharpiro–Wilk test.

**Table 3:** Changes in clinical symptoms: dep., somatic, and anxiety (outlier included: *N* = 7).

	Pretest	Posttest	*p*-value for model	Effect sizeCohen’s *d*
PHQ-9 (Depression Symptoms)	8.57 ± 5.01	7 ± 6.85	0.2 (NS)	–
PHQ-15 (Somatic Symptoms)	10.71 ± 4.83	9.2 ± 5.3	0.18 (NS)	–
GAD-7 (Anxiety Symptoms)	7.4 ± 4.9	6.86 ± 4.12	0.41 (NS)	–

*Decreased scores on all clinical symptom measures indicate improvement*.

*Significantly decreased between pretest and posttest p ≤ 0.10.

**Significantly decreased between pretest and posttest p ≤ 0.05.

***Significantly decreased between pretest and posttest p ≤ 0.01.

*NS, non-significant results*.

### PHQ-SADS Graphic Display of Data (Outlier Included)

**Figure 3:**
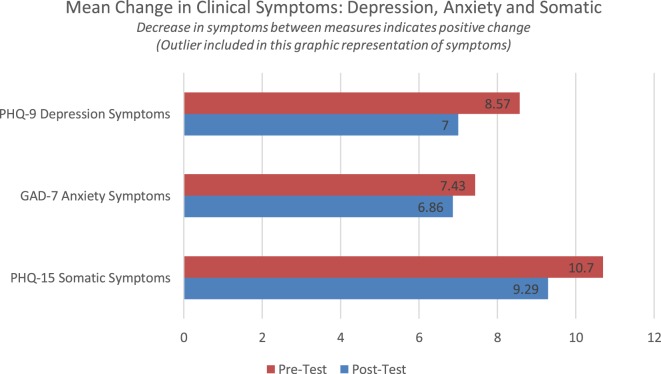
Change in clinical symptoms: depression, anxiety, and somatic.

These data indicated a modest reduction in clinical symptoms between the first and second measurement point (Figure 3). Changes in depression and somatic symptoms would indicate clinical change that could be felt by the participant and observed by a clinician. None of the clinical symptom profiles indicate a statistically significant change between pretest and posttest. Depression symptoms were reduced by 1.57 points, anxiety by 0.57, and somatic symptoms by 1.28 points.

### PHQ-9, GAD-7, and PHQ-15 (With Outlier Excluded)

One member of this group was found to likely be an outlier in using the Dixon outlier test with *p*-value of with 0.024, 0.028, and 0.006 compared to each of the above measures posttests. The data associated with this participant also ranged from 2 to 3 SDs higher the mean. There were several notable factors when comparing this individual case to others that may indicate why this individual does not fit with the group mean. These are higher level of reported illness including chronic pain, insomnia, and other chronic health conditions. This individual also had a higher level of psychosocial stressors than others reporting a history of homelessness and higher levels of exposure to violence. It may be that this individual case would represent an interaction between the impacts of gender identity and chronic illness. In further studies adapting the group materials to address chronic illness could impact this outcome.

Due to the above factors, it appeared worth considering the data set without this case included. The data without this case included trended in both a more clinically meaningful and statistically significant direction. With this outlier excluded data trended toward significant reduction in symptoms of depression and somatic symptoms with *p* = 0.093 and 0.097, respectively. These data had a modest effect size using the Cohen’s *d* test. The findings indicated effects size of *d* = 0.68 for depression symptoms and *d* = 0.72 on somatic symptoms. Anxiety symptoms were not found to be reduced significantly. Data sets shown in Table 4 continued to meet criteria for normality after removal of outlier using visual assessment of line on Q–Q plot as well as Sharpiro–Wilk test.

**Table 4:** Changes in clinical symptoms: dep., somatic and anxiety (outlier excluded *N* = 6).

	Pretest	Posttest	*p* for model	Effect sizeCohen’s *d*
PHQ-9 (depression symptoms)	7 ± 3.4	4.3 ± 2.4	0.093*	0.68
PHQ-15 (somatic symptoms)	9.5 ± 3.98	7.33 ± 2.36	0.097*	0.72
GAD-7 (anxiety symptoms)	7 ± 5.3	5.3 ± 1.9	0.27 (NS)	–

*Decreased scores on clinical symptom measures indicate improvement*.

*Trending toward significant decreased between pretest and posttest p ≤ 0.10.

**Significantly decreased between pretest and posttest p ≤ 0.05.

***Significantly decreased between pretest an posttest p ≤ 0.01.

*NS, non-significant results*.

### PHQ-SADS Graphic Display of Data (With Outlier Excluded)

**Figure 4:**
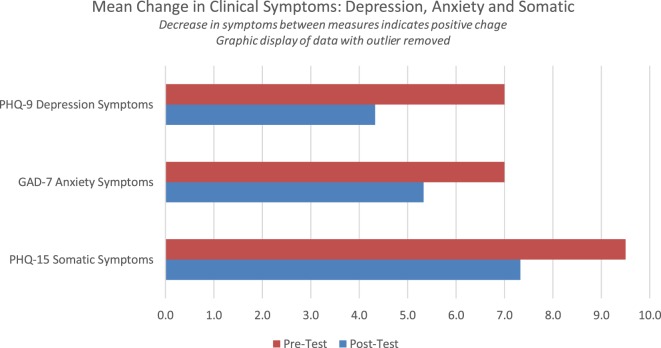
Mean change in clinical symptoms depression, anxiety, and somatic symptoms.

Reduction of clinical symptoms between pretest and posttest indicates an improvement in mental health. Figure 4 shows changes in symptoms of depression with one case identified as an outlier removed. Reduction in depression symptoms and somatic symptoms between first and final measure that trend towards significant reduction with *p* = 0.093 and 0.097, respectively, with a moderate effect size of *d* = 0.68; and *d* = 0.72, respectively. Symptoms of anxiety did not show significant reduction between measures. Depression symptoms were reduced by 3.6 points between measures, somatic symptoms were reduced by a mean of 2.17 points, and anxiety symptoms were reduced by a mean of 1.7 points. These changes would indicate a reduction in clinical symptoms that likely could be observed by both a clinician and would be experienced as meaningful by the participant in the study.

### *Post Hoc* Analysis Findings

#### *Post Hoc* Analysis Clinically Significant Depression Symptoms (PhQ-9)

**Figure 5:**
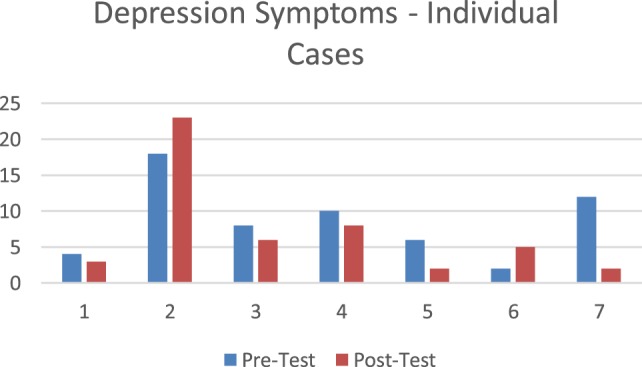
Individual change in depression symptoms.

Two participants who completed the PHQ-9 were in the clinically significant range. Over the course of the treatment these individuals moved below the clinically significant range moving from 10 to 8 (moderate depression symptom score to mild/no depression score) and 12 to 2, respectively (moderate depression symptom score to mild/no depression score). Two individuals had a modest increase in symptoms, with one individual having a 5 point increase shifting into the severe range and another an increase of 3 points remaining in the minimal to no symptom range. Figure 5 displays pretest posttest data for individual participants’ depression symptoms.

#### *Post Hoc* Analysis Clinically Significant Anxiety Symptoms (GAD-7)

On the measure of anxiety symptoms (GAD-7), there were two individuals that scored in a range that is considered likely to indicate the presence a clinical diagnosis of anxiety. Their scores were 11 and 15, respectively. These individuals’ range had what would have been a meaningful reduction in their clinical symptoms moving from 11 to 2 (moderate symptom score range to mild/none score range) and 15 to 5 (severe symptom score range to mild/no symptom score range) on the GAD-7.

Those in the subclinical range had no significant reduction, either staying the same or displaying a modest increase staying in the mild to no symptom range. Three individuals had an increase in anxiety symptoms, these ranged from a 4 to 6 point increase. Two of these individuals had near 0 initial symptoms and moved to a score of 5 (from no symptoms to no to minimal symptoms). One individual had an increase in anxiety from a score of 10 (mild anxiety) to a score of 16 (moderate anxiety range).

**Figure 6:**
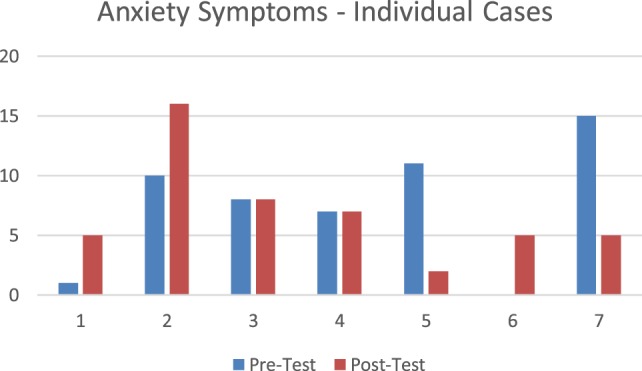
Individual change in anxiety symptoms.

It is possible that increased somatic awareness increased their awareness of the physiological stress that they were experiencing. The individual who showed the highest level of increase also was in the group with the highest rate of health and mental health conditions reported, the highest exposure to gender-based violence, and had experienced significant life stressors. This might indicate a need for increased focus on chronic conditions and self-care in future adaptations of the curriculum. Figure 6 displays pretest posttest data for individual participants’ anxiety symptoms.

#### *Post Hoc* Analysis Clinically Significant Somatic Symptoms (PHQ-15)

**Figure 7:**
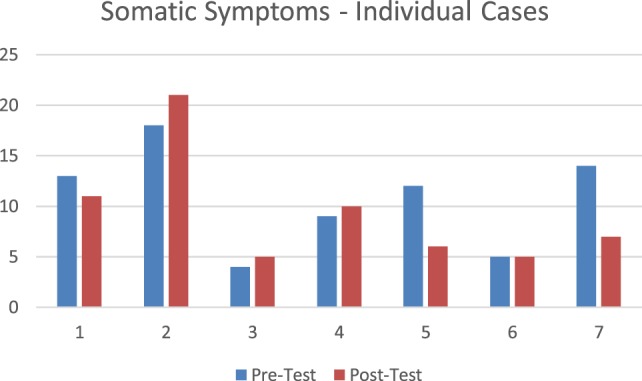
Individual change in somatic symptoms.

Three participants had a reduction in symptoms. Two of these individuals moved from moderate symptoms to mild to no symptom range. These individuals had a 7-point and a 6-point reduction, respectively. And the other individual who showed symptom reduction went from high mild symptoms to low mild symptoms (2-point symptom reduction). Three individuals had an increase in scores. Two displayed a modest increase of 1 point between each measure and one participant had a 5-point increase in score. The individuals who had increased scores all had higher levels of overall health concerns, two reported three incidents of exposure to violence related to their gender identity and all three had experienced homelessness. Figure 7 displays pretest posttest data for individual participants on somatic symptoms.

#### *Post Hoc* Analysis Coping with Discrimination Scale (CDS)

There were four individuals who completed the CDS and all other measures. Two of these individuals had an increase in sub-scale on CDS that measures coping through education (a positive empowered form of coping). For these participants, there were larger reduction in symptoms on all levels of SADS-PHQ than those with reduced education score on CDS.

### Participant Race, Ethnicity, Biological Sex, Gender Identity, and Self-Description

A total number of eight individuals were screened as appropriate candidates to participate in the SE™ informed group study. One individual later dropped out from the study, and we will report responses of all eight individuals. All eight identified as “white.” One participant also identified as “American Indian/Alaska Native,” while another also identified as “Hispanic/Latino/Latina.”

Of the eight participating individuals, all considered themselves to be transgender/gender non-conforming/gender variant/gender diverse in some way. Five were born biologically female and three were born biologically male, as indicated on their original birth certificate. In response to “what is your primary gender identity today,” one biologically born female identified as “male/man,” two biologically born males identified as “female/woman.” Other self-identified gender descriptions included: “non-binary demi-boy” (biologically born female, also group drop-out), “no gender trans-female” (biologically born male), “non-binary, gender queer” (biologically born female), “no gender, some male/some female” (biologically born female), “unknown” (biologically born female).

Participants were given the opportunity to respond to questions regarding whether they were in transition from a M → F or female to male (F → M) binary gender identity, and whether any were receiving medically monitored HRT. Seven respondents indicated that they were in transition. One (the group’s drop-out) was unclear in their gender transition response, had completed desired transitioning, and was not receiving HRT. A F → M indicated that they had not completed desired transitioning, and they were receiving HRT. Another F → M indicated that they had not completed desired transitioning, and they were not receiving HRT. A M → F indicated that they had completed desired transitioning, and they were receiving HRT. Another, who considered themselves F → M → FUnsure, had completed desired transitioning and was receiving HRT. Another who considered themselves F →?, had not completed desired transitioning, and was not receiving HRT. Another participant, who considered themselves non-binary, had completed desired transitioning and was not receiving HRT. One last respondent, who was ambiguous (answered yes and no) regarding whether they were in the process of transitioning, considered themselves “res binary,” had completed desired transitioning, had received top surgery and oophorectomy, and was not receiving HRT. Of the three participants indicating that they were receiving HRT, none were obtaining hormones from the internet.

Participants were asked to respond with “not at all,” “somewhat,” or “strongly” to a listing of self-describing terms used in the Transgender community. Responses are presented in Table 5: Group Participants’ Self-Describing Gender Terms.

**Table 5:** Group participants’ self-describing gender terms.

Self-describing terms	Not at all	Somewhat	Strongly	No answer
Transexual	1	4	2	1
MtF (Male to Female)	3	1	2	2
FtM (Female to Male)	2	4	1	1
Intersex	7			1
Gender variant/gender NON-conforming		1	7	
Gender fluid	4	3	1	
Genderqueer		4	4	
Androgynous	3	4	1	
Feminine male	7	1		
Masculine female or butch	3	5		
A.G. or Aggressive	7			1
Third gender	2	5	1	
Cross dresser	8			
Drag performer (Queen/King)	7	1		
Two-spirit	5	2	1	
		
Other, please specify	Demi-boy, A-gender queer, femme, non-binary			

### Family Composition, Living Arrangement, Supports

Determination of support systems, relationships, living arrangements, employment, school, and homelessness were explored. All eight respondents reported having a circle of friends or family that they could count on. Five participants identified as “single.” Of these five, one respondent was employed, a student, had never experienced homelessness, and lived with their three children in a home that they owned. All of the remaining participants reported having no children. Another single respondent was not a student, not employed, had previously experienced homelessness, currently lived with a roommate/friend, and paid rent. A third single respondent was not a student, was employed, lived alone, had experienced homelessness in the past, and did not answer as to whether they owned or rented. The fourth single participant had responded as both single and partnered, they were not a student, not employed, they had experienced homelessness in the past, they currently lived with a roommate/friend, and they paid rent. The final single respondent was not a student, was not employed, had never experienced homelessness, they currently lived with a roommate/friend, and they paid rent.

The remaining three of eight respondents identified as “partnered.” One of them was not a student, was not employed, had previously experienced homelessness, lived with a spouse/partner/significant other, and paid rent. Another partnered respondent was a student, was employed, had never experienced homelessness, lived alone, and owned their home. The third partnered respondent also identified as having a non-monogamous relationship, was a student, was employed, had never experienced homelessness, lived with a roommate/friend, and paid rent.

### Health Factors

Participants were polled for having some common medical conditions. None reported having diabetes, heart problems, nor kidney disease. One reported having high blood pressure that was controlled with prescribed medication.

Three study participants reported having a formal diagnosis of Gender Dysphoria. All three also saw a mental health provider for psychotherapy support, two of the three took prescribed medication for a mental health condition, and one of these two also saw a psychiatrist for medication management. A fourth participant was not sure if they had a formal Gender Dysphoria diagnosis but took prescribed medication for a mental health condition. The remaining four participants responded as not having a diagnosis of Gender Dysphoria. Only one of these four saw a mental health provider, and another one of the four took herbal supplements for mental health. Degrees of experiences of depression, anxiety, anger/irritability, difficulty sleeping, and pain were also solicited (see Table 6).

**Table 6:** Responses to types of experiences with some mental health conditions.

Mental health conditions	Do not experience	Intermittent/occasional	Constant, frequent or chronic	No answer
Depression	1	3	4	
Anxiety	2	3	2	1
Anger/irritability	1	5	2	
Difficulty sleeping	5	2	1	
Pain	1	4	3	

Active substance use was explored. None of the respondents acknowledged cocaine, methamphetamine, or non-prescribed benzodiazepine use. Of eight respondents, two used cigarettes or tobacco products, while six did not. Of the two smokers, one consumed alcohol on a daily basis, while the other did not. One other participant consumed only alcohol on a daily basis. Three respondents acknowledged using only cannabis ranging from daily to monthly, while the remaining five did not. Two of the remaining respondents reported using no alcohol, tobacco, or other illicit substances whatsoever.

### Identified Barriers to Health Care

Barriers to routine, preventative, or emergency health care were examined. Routine health care would include cervical exams, breast exams, prostate exams, accidents, etc. Two of the eight respondents did not try to access any of these types of health care, giving reasons as financial/no insurance and discomfort about meeting with health-care providers as barriers. Another respondent gave financial/no insurance as a barrier, and two others gave discomfort about meeting with health-care providers as a barrier. Another respondent gave discrimination/abuse/mistreatment by providers as an only barrier, while the two remaining participants indicated “none of these” as a response.

Of eight survey participants, three acknowledged having no desire or have not decided to access reconstructive, enhancement, or gender reaffirming surgeries. Another three of the eight respondents gave reasons of financial/no insurance as barriers against accessing these services. Another responded that finances or lack of insurance, discrimination, abuse, mistreatment by providers, and discomfort about meeting with health-care providers were all barriers. One responded that they could not locate a Medicare-funded surgeon for desired procedures.

### Experiences of Mistreatment, Violence, Abuse, and Harassment

Survey participants were asked to respond to questions related to several different specific experiences of mistreatment, violence, abuse, harassment. Five reported no mistreatment (verbal abuse, disrespect, harassment) from health-care providers because of their gender identity, while two acknowledged some form of mistreatment, and one did not provide an answer. Four participants denied experiencing any form of partner or domestic related abuse or violence related to their gender identity. Four acknowledged some form of partner, domestic related, or other violence in the past relative to their gender identity. Five of eight respondents acknowledged current verbal abuse or harassment in their day to day activities, while three did not. Five of eight respondents acknowledged other current or past types of harassment or abuse in their day to day activities, one did not, one did not because they were not “out,” while one did not answer.

## Discussion

The exposure to experiences of discrimination can have a highly negative effect on health and mental health. Some previous studies have identified ANS functioning and long-term exposure to stress hormones as the mediating factor between stress, health and discrimination. Dysregulation in the ANS has been found to have multiple long-term health and mental health impacts. ANS dysregulation may be one of the moderating if not mediating factors that lead to increased rates of depression, suicide and mortality in individuals who identify as transgender or gender fluid. Increased, resilience and coping could improve ANS functioning. Developing skills that support improved resilience, increased ANS regulation and develop capacity to cope with experiences of discrimination could improve health, mental health, and QoL/well-being.

These data indicated a modest clinically impactful and statistically significant improvement in one marker of resilience for individuals who identify as gender fluid and gender non-conforming, e.g., psychological QoL. Data showed significant increases in psychological QoL (a measure of mental wellbeing) (*p* < 0.004). Increased psychological wellbeing would have a meaningful impact on the ability to be resilient after a stressor and to psychological adapt to challenges.

The skills taught in the group focused on increasing somatic awareness and mindfulness and the ability to regulate difficult emotions. These skills could have an impact on the capacity to manage the many stressful events in the lives of individuals’ ongoing exposure to discrimination and threat of community violence. It is notable that just prior to the final assessment one of the most violent assaults to the LGBTQIA community in US history occurred. Forty-nine individuals from the LGBTQIA community were killed and 53 people were injured at the Pulse nightclub in Orlando, FL, USA. The psychological impact was likely significant. However, this group still displayed increased psychological well-being overall.

When considering the entire data set there were no significant decreases found in mental health symptoms. The data would have, however, indicated a modest improvement in overall mental health that may have been able to be felt by the participants and observed by a clinician.

Data were analyzed to assess for outliers and an outlier was identified. When reviewing factors that may have driven the increased initial and follow-up symptoms, this participant had a higher rate of chronic illness and psychosocial stressors. Other group treatment modalities have solved these problems by adapting the group materials to address increased support and case management as well as to address complex health issues. These could be addressed in later adaptations of this group.

It also appeared that exploring these data with this data point removed would offer some meaningful insights. When this outlier case was removed the data showed an increase in the amount of clinical change into a more meaningful clinically significant range. The data also indicated significant decreases in symptoms of depression (*p* < 0.093) and somatic symptoms (*p* < 0.097). These symptom reductions could indicate a reduction from moderate symptoms to minimal symptoms. This would translate to increased ability to effectively engage with work, relationships, and enjoyment of life.

This study offers initial indication that a resiliency-based SE™ informed group therapy intervention model for reducing negative symptoms of Gender Dysphoria could increase markers of resilience. Future adaptions in the group materials can take into account findings related to chronic illness and life stressors. While the data have significant limitations, it provides a demonstration of the potential efficacy of a therapeutic group model that can be repeatedly replicated, applied and used to help Transgender, Gender-Non-Conforming and Gender-Variant persons within communities.

With tools and self-awareness gained, participating Transgender, Gender-Non-Conforming and Gender-Variant persons may become more hopeful and resilient to better manage the multiple challenges that inherently exist through experienced psychosocial barriers, discrimination, and social injustice. Intrinsically, injustice does not go away easily and on its own, and will, unfortunately, remain a strong influential factor in the lives of Transgender, Gender-Non-Conforming, and Gender-Variant persons.

The hope is that this group can help participants to feel less victimized and more self-empowered, to become stronger self-advocates, to become more educated, to build resiliency, and to build unity and decrease isolation. Some have argued that exposure to discrimination and community violence represent a traumatic event in and of itself. These data point toward the possibility of developing capacity to address the impact of multiple forms of discrimination and increased health and mental wellbeing for individuals exposed to toxic interpersonal experiences.

Relative to his therapeutic work with Vietnam Veterans, Bessel van der Kolk (49) observed that Veterans in a group setting could speak with the intensity of traumatic combat experiences, as they found resonance and meaning among the sharing of experiences of other Veterans. Van der Kolk observed a renewed sense of comradeship, vital during their service experiences. “You were either in or out- you either belonged to the unit, or you were nobody, the world is divided between those who know and those who don’t know, people who have not shared in a traumatic experience are not to be trusted because they cannot understand it.” This phenomenon was noted to occur in our Somatic Experiencing^®^ Informed Trans Support and Healing Group. Unlike van der Kolk’s Veterans’ group, who could not make a connection between their wartime experiences and behaviors in their current lives, our group was able to demonstrate the experience of more of a connection.

### Limitations

Posttest follow-up measures were conducted during the week following the June 12, 2016 fatal shooting massacre at Pulse Orlando, a gay nightclub in Orlando, FL, USA. Forty-nine people were killed and 53 were injured. This horrific act was portrayed in the media for several weeks, creating a sense of fear, sadness, anxiety, as well as of solidarity in the LGBTQIA community worldwide. We are certain that this event had a significant influence on group participants’ anxiety and stress levels, and their feelings and perceptions of overall safety at the time of post evaluation.

Our group size was relatively small, and the entire demographic make-up of race and ethnicity consisted of eight white participants, with one participant also identifying as “American Indian/Alaska Native,” and another also identifying as “Hispanic/Latino/Latina.” Diversity inclusive of people of color and other races and ethnicities would be a factor of great value for future studies.

### Future Directions of Research

Further research needs to be done in a larger sample with randomization. This line of research has a real potential to improve the lives of the transgender/gender non-conforming community. More research is needed for the exploration of how SE™ and other trauma-based treatments can address social trauma, which is chronic and persistent, often for a lifetime, in comparison to traumatic events which have no social component, have happened in the past, and which are often time-limited in nature.

As the literature develops it is possible that this model will be transferable for use in the care and treatment of the effects of social injustice upon other oppressed and marginalized populations. With minor changes, the therapeutic group curriculum can be modified to fit the needs of other specific target populations, or else generalized to target a wider population. SE™ informed segments, such as ANS regulation, social engagement, resourcing, and safety, would remain universal within the curriculum as the foundation of the therapeutic group work. Therapeutic goals would also remain universal as: a general increase in awareness of resources, skills and abilities, increased skills for affect management, increased skills for nervous system regulation, increased capacity for tolerance of “being in their bodies” and managing “real life” situations relating to confrontations and microaggressions, increased resiliency, a decrease of negative symptoms such as depression, anxiety and feelings of social isolation.

## Author Contributions

PB is principal investigator. He developed protocol for group intervention in collaboration with SH. He wrote multiple sections of the article and compiled all data. SH conducted the group treatment. They developed the treatment protocol in collaboration with PB. They consulted on issues related to the community where the study was conducted. MC was a consultant on the project. He supported IRB document development, research protocol development, made recommendations about outcomes measures, and conducted data analysis and supporting paper write up and submission.

## Conflict of Interest Statement

PB is an SE™ practitioner (SEP) who derives income from his practice. SH is an SE™ practitioner (SEP) who derives income from their practice and is a training assistant with the Somatic Experiencing Trauma Institute™. MC is a member of the board of directors for Somatic Experiencing Trauma Institute™, who receives a small yearly stipend for that work, and chair of SE™ research coalition, an unpaid position.
